# A Novel Immune-Prognosis Index Predicts the Benefit of Lung Adenocarcinoma Patients

**DOI:** 10.3389/fphar.2022.818170

**Published:** 2022-05-09

**Authors:** Yuquan Bai, Yun Pei, Liang Xia, Lin Ma, Senyi Deng

**Affiliations:** ^1^ Department of Thoracic Surgery and Institute of Thoracic Oncology, West China Hospital, Sichuan University, Chengdu, China; ^2^ Department of Interventional Radiology and Vascular Surgery, Peking University Third Hospital, Beijing, China

**Keywords:** lung adenocarcinoma, immune cell infiltration, immune checkpoint blockade (ICB), immune-prognosis index (IPI), network analysis

## Abstract

**Background:** Constructed an immune-prognosis index (IPI) and divided lung adenocarcinoma (LUAD) patients into different subgroups according to IPI score, describe the molecular and immune characteristics of patients between different IPI subgroups, and explore their response to immune checkpoint blockade (ICB) treatment.

**Methods:** Based on the transcriptome profile of LUAD patients in TCGA and immune gene sets from ImmPort and InnateDB, 15 hub immune genes were identified through correlation and Bayesian causal network analysis. Then, IPI was constructed with 5 immune genes by using COX regression analysis and verified with external datasets (GSE30219, GSE37745, GSE68465, GSE126044 and GSE135222). Finally, the characteristics and the response to ICB treatment of LUAD patients between two different IPI subgroups were analyzed.

**Results:** IPI was constructed based on the expression of 5 genes, including A2M, ADRB1, ADRB2, VIPR1 and PTH1R. IPI-high LUAD patients have a better overall survival than IPI-low LUAD patients, consistent with the results in the GEO cohorts. The comprehensive results showed that patients in the IPI-high subgroup were exhibited characters as metabolism-related signaling pathways activation, lower TP53 and TTN mutation rate, more infiltrations of CD8 T cells, dendritic cells and macrophages M1, especially earned more benefit from ICB treatment. In contrast, patients in the IPI-low subgroup were exhibited characters as p53 signaling pathways activation, higher TP53 and TTN mutation rate, more infiltrations of resting memory CD4 T cells, macrophages M2, immune-suppressive response and less benefit from ICB treatment.

**Conclusion:** IPI is a potentially valuable prognostic evaluation method for LUAD, which works well in the benefit predicting of LUAD patients within ICB treatment.

## 1 Introduction

Immune checkpoint blockade (ICB) treatment is designed to attack cancer cells by activating the immune system (Mahoney et al., 2015). As targeted programmed death-ligand 1 (PD-L1), programmed death 1 (PD-1) and cytotoxic T lymphocyte-associated protein 4 (CTLA4), the existed ICB treatment has significantly improved the overall survival (OS) of patients in several cancers (Larkin et al., 2015; Motzer et al., 2015; Bellmunt et al., 2017). In advanced LUAD, patients within nivolumab treatment could earn significantly benefits, both in the remission rate, OS and progression-free survival (PFS) (Brahmer et al., 2015). However, low response rate which limited LUAD patients survival from ICB treatment extremely (Sharma et al., 2017). In fact, many issues could influence the therapeutic effect of ICB treatment, including immune cell infiltration (Van Allen et al., 2015; Nishino et al., 2017), PD-L1 expression (Patel and Kurzrock 2015), tumor mutation burden (TMB) (Samstein et al., 2019), tumor antigen presentation (Snyder et al., 2014; Zaretsky et al., 2016) and so on. But evaluation method based on these issues still cannot make an effective prediction in LUAD patients after ICB treatment (Nishino et al., 2017). Therefore, it remains needed for us to identify new prognostic biomarkers which could indicate the response of ICB treatment in LUAD, also explore novel evaluation method by using such biomarkers.

In this study, we first isolated the differential immune genes in the LUAD patients (tumor *vs*. normal lung tissue), and used correlation and causal network analysis to get hub immune genes, then identified genes which significant related to the prognosis of LUAD, and finally construct the immune-prognosis index (IPI). We tested the efficient of IPI in prognosis prediction, demonstrated that IPI could predict the prognosis of LUAD patients both in conventional and immune therapy. We also analyzed the molecular and immune characteristics between different IPI subgroups, which further indicated that the efficient of IPI in prognosis prediction was based on realizing the anti-tumor immune status in patients.

## 2 Materials and Methods

### 2.1 Patients and Datasets

The transcriptome data of LUAD samples (535 tumors *vs*. 59 normal lung tissues) and clinical information were downloaded from the TCGA database (https://portal.gdc.cancer.gov/). The transcriptome data and survival information of the validation data sets (GSE30219 (293 tumor samples), GSE37745 (196 tumor samples), GSE68465 (442 tumor samples)) were downloaded from Gene Expression Omnibus (https://www.ncbi.nlm.nih.gov/geo). The immune gene sets were downloaded from the ImmPort (https://www.immport.org/shared/home) and InnateDB (https://www.innateDBdb.com/) databases.

### 2.2 Correlation and Causal Network Analysis to Identify Differential Immune Genes

Based on the expression profile data of LUAD in TCGA, we used the “limma” R package to get the differentially expressed genes (*p*-value < 0.05, |log2FC| > 1.5). Intersecting the obtained differential genes with the immune genes obtained from ImmPort and InnateDB, we finally got the differential immune genes. The obtained differential immune genes were used for functional enrichment analysis with Gene Ontology (GO) and Kyoto Encyclopedia of Genes and Genomes (KEGG).

Then, Weighted Gene Co-expression Network Analysis (WGCNA) was used to determine the LUAD-related immune gene modules (Langfelder and Horvath 2008). According to abline = 39 and *β* = 5 as the soft threshold, WGCNA was used to construct a weighted gene co-expression network. Finally, removing the grey module, we got two modules, and the minimum number of genes in each clustering was set 30. According to the heatmap of module-trait relationships, we found the blue and turquoise modules were significantly correlated with LUAD. Two modular gene were subjected to functional enrichment analysis using “clusterProfiler” R package (v3.9.2; https://github.com/GuangchuangYu/clusterProfiler). Next, “STRINGdb” R package was used to calculate the interaction of these modular genes (Szklarczyk et al., 2015). According to the weight >900, gene-pairs with strong interaction in the blue and turquoise modules were obtained. Then, screened by degree >5, 44 co-expressed genes were identified (Supplementary Table S3C).

Finally, Bayesian causal network analysis was used to determine immune hub genes of LUAD (Puga et al., 2015). We used the “bnlearn” R package to construct a Bayesian causal network (200 iterations) between the 44 differential immune genes identified above. Screening based on weight >0.8, we identified 39 genes and 79 gene-pairs (Supplementary Tables S4A, B), and used Cytoscape (3.7.2) to draw this causality network. Then screened by degree ≥5, and finally 15 immune hub genes were determined. We finally analyzed the biological functions and gene mutations of 15 immune hub genes.

### 2.3 Construction and Verification of Immune-Prognosis Index

Through univariate COX regression analysis, we identified 5 immune-prognosis genes. The “survival” and “survminer” R packages were used for further survival analysis. The Kaplan-Meier method was used to estimate the survival curve, and the log-rank test was used to analyze the difference in survival time. At the same time, we analyzed the network relationships and biological functions of 5 genes, and mapped the network relationships by Cytoscape (3.7.2).

Five immune-prognosis genes that significantly affect the prognosis of LUAD were used to construct a prognosis model through multivariate COX regression analysis. 504 LUAD samples with survival information were used as the training set, and GSE30219 (274 LUAD samples), GSE37745 (196 LUAD samples) and GSE68465 (442 LUAD samples) were used as the validation set. The calculation formula was as follows: risk score = Σ(C × EXP_RNA_). In our formula, EXP_RNA_ represents the expression of 5 IPI genes, C represents the corresponding coefficient of multivariate Cox proportional hazards regression. To validate the independent prognostic value of IPI, univariate and multivariate COX regression analysis was performed.

### 2.4 The Molecular, Immune Characteristics and the Response of anti-PD-1 Therapy in IPI Subgroups

We compared whether the distribution of clinical characteristics between IPI subgroups was even. Next, we performed GSEA and mutation analysis to compare the biological functions and molecular characteristics between IPI subgroups. In order to determine the composition of immune cells in the LUAD samples, we imported the expression of LUAD into CIBERSORT (https://cibersort.stanford.edu/) (iterated 1000) (Newman et al., 2015), and finally obtained the relative proportions of 22 immune cells. We analyzed the difference and correlation between 22 immune cells in different IPI subgroups. In addition, we also analyzed the correlation analysis of PD-1/PD-L1, CTLA4 and HLA-E and IPI scores.

To further determine the immune and molecular functions between IPI subgroups, we performed ssGSEA analysis on certain gene signatures and compared the scores between the two IPI subgroups. At the same time, we draw survival curves for immune and molecular functions (*p* < 0.05) between IPI subgroups.

In order to determine the relationship between IPI subgroups and clinical characteristics and reported immunotype, we grouped LUAD patients according to tumor stage, pathological subtype, and reported immunotype, and analyzed their interactions.

In order to explore the prognostic value of IPI in ICB treatment, we conducted survival analysis on GSE126044 (n = 9) and GSE135222 (n = 27) cohorts of LUAD patients receiving anti-PD-1 therapy. In addition, we compared the area under the curve (AUC) of IPI, TIDE, TIS and two external models at 3-years of follow-up to determine the prognostic value of different models. Tumor Immune Dysfunction and Exclusion (TIDE) score was calculated online (HTTP://tide.dfci.harvard.edu/). T-cell-inflamed signature (TIS) is an 18-gene signature including genes which reflect the response of an ongoing adaptive Th1 and cytotoxic CD8 T cell in anti-PD-1/PD-L1 therapy (Ayers et al., 2017). Two articles on immune signature models predicting prognosis and responsiveness to ICI therapy in LUAD patients were used to validate the accuracy and specificity of IPI model (Guo et al., 2020; Yi et al., 2021).

## 3 Results

### 3.1 Correlation and Causal Network Analysis to Identify Differential Immune Genes in Lung Adenocarcinoma

**FIGURE 1:**
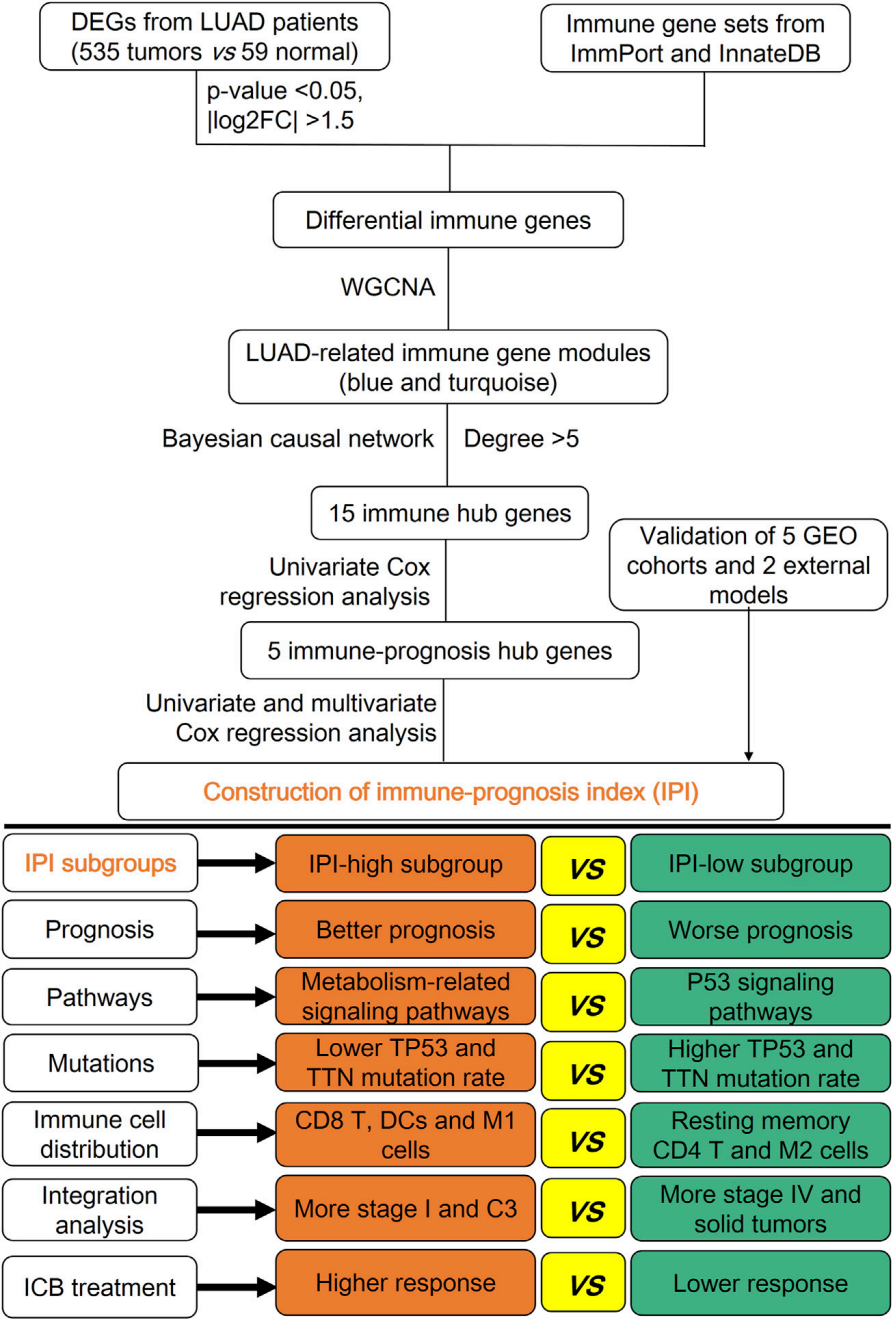
Graphical abstract for the construction, verification and characterization of IPI in LUAD.

The graphical abstract for the construction, verification and characterization of IPI in LUAD is depicted in Figure 1. In the differential expression analysis between 535 tumors *vs*. 59 normal lung tissues of LUAD patients, we obtained 3764 differential genes, including 2747 up-regulated genes and 1017 down-regulated genes (Supplementary Figure S1A; Supplementary Table S1). Intersecting these genes with immune gene sets obtained from ImmPort and InnateDB, 437 differential immune genes were obtained, including 264 up-regulated genes and 173 down-regulated genes (Supplementary Figure S1B). The functional analysis of 437 differential immune genes were enriched in 1516 GO terms and 33 KEGG pathways, and we showed the top 20 GO terms and KEGG pathways (Supplementary Figure S1C,D; Supplementary Table S2A,B).

In order to determine the gene modules related to LUAD, WGCNA was used to perform co-expression analysis of 437 candidate differential immune genes. The optimal soft-thresholding power was 5 based on the scale-free network. Then based on the optimal soft-thresholding power and the average linkage hierarchical clustering, three modules were finally determined (Supplementary Figure S2A). According to the Pearson correlation coefficient between gene modules and samples, we found that the blue (49 genes) and turquoise (204 genes) modules were correlated with LUAD (Supplementary Figure S2B), and these genes were used for subsequent analysis. The top eight GO terms and KEGG pathways of genes in the blue and turquoise modules were respectively shown (Supplementary Figure S2C,D; Details in Supplementary Tables S2C–F). Screened with the threshold of weight >900, we showed the correlation network of blue (29 gene-pairs) and turquoise (226 gene-pairs) modules (Supplementary Figure S2E,F; Supplementary Table S3A,B). Then screened according to the degree >5 of correlation network genes, we finally identified 44 genes (Supplementary Table S3C). After intersecting 44 genes with five GEO datasets, 41 immune-related genes were finally identified, and batch correction was performed using the ComBat algorithm in the all samples.

Next, based on the expression of LUAD in TCGA and the gene strength >0.8 in the Bayesian causal network, we got 39 genes and 79 gene-pairs (Supplementary Figure S3A; Supplementary Table S4A). Then according to the degree ≥5, 15 immune hub genes were finally obtained (Supplementary Table S4B). Most of these immune hub genes were enriched in G protein-coupled receptor signaling pathway, cell chemotaxis and IL-17/TNF signaling pathway (Supplementary Figures S3B,C). Meanwhile the mutation rate of these immune hub genes was less than 5% (Supplementary Figure S3D).

### 3.2 Construction and Verification of Immune-Prognosis Index in Lung Adenocarcinoma

**FIGURE 2:**
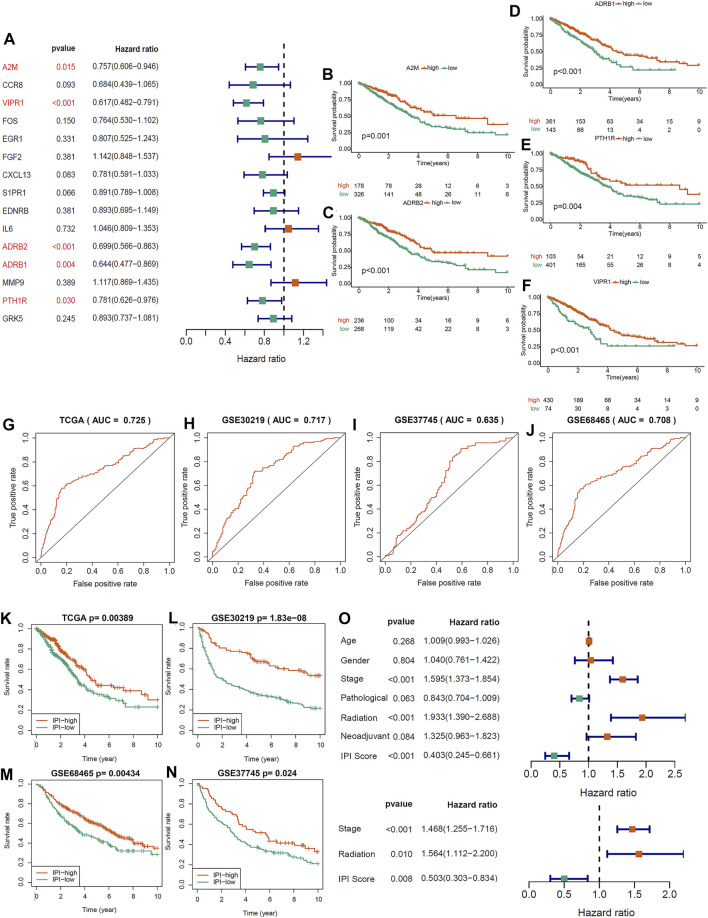
Constructing an immune-prognosis index (IPI) for LUAD. **(A)** Univariate COX regression analysis was used to determine the prognostic-related genes in 15 immune hub genes. **(B–F)** Kaplan-Meier survival analysis of 5 prognostic-related genes (*p* < 0.05). **(G–J)** ROC analysis of the IPI in the LUAD of TCGA and GEO cohorts. **(K–N)** Kaplan-Meier survival analysis of the IPI subgroups in the LUAD of TCGA and GEO cohorts (*p* < 0.05). **(O)** Univariate and multivariate COX regression analysis to determine the independent prognostic value of IPI in LUAD.

In order to determine immune-prognosis genes, we performed univariate COX regression analysis on 15 immune hub genes. Only 5 genes (A2M, ADRB1, ADRB2, VIPR1 and PTH1R) were significantly affected the outcome of LUAD patients (all HR < 1), and the Kaplan-Meier survival curves of these genes were drawn (Figures 2A–F). Besides, we analyzed the correlation and biological functions between 5 immune-prognosis hub genes and immune genes obtained from ImmPort and InnateDB. We found that these genes were mainly enriched in cAMP-mediated signaling, G protein-coupled receptor signaling pathway and cAMP/MAPK signaling pathway (Supplementary Figure S4A–C, Supplementary Table S5). Then, we constructed a prognostic index for the LUAD patients, using the formula IPI = (−0.0125 × EXP_A2M_) + (0.3224 × EXP_VIPR1_) + (0.22 × EXP_ADRB2_) + (0.1663 × EXP_ADRB1_) + (0.0329 × EXP_PTH1R_). Grouping by the median of IPI score, we found that the AUC and 5-years survival rate of LUAD patients in the IPI-high subgroup was significantly higher than IPI-low subgroup (AUC = 0.725, *p* = 0.00389, Figures 2G,K). At the same time, we also got this result in three validation datasets (all *p* < 0.05; Figures 2H–J; Figures 2L–N).

Between IPI subgroups, these clinical characteristics were distributed equally (Supplementary Figure S5). Univariate COX regression analysis showed that IPI, tumor stage and radiation therapy were significantly related to the prognosis of LUAD (Figure 2O). Moreover, multivariate COX regression analysis showed that IPI could act as an independent prognostic factor after adjusted other clinical characteristics (Figure 2O).

### 3.3 Molecular Characteristics of Immune-Prognosis Index Subgroups

**FIGURE 3:**
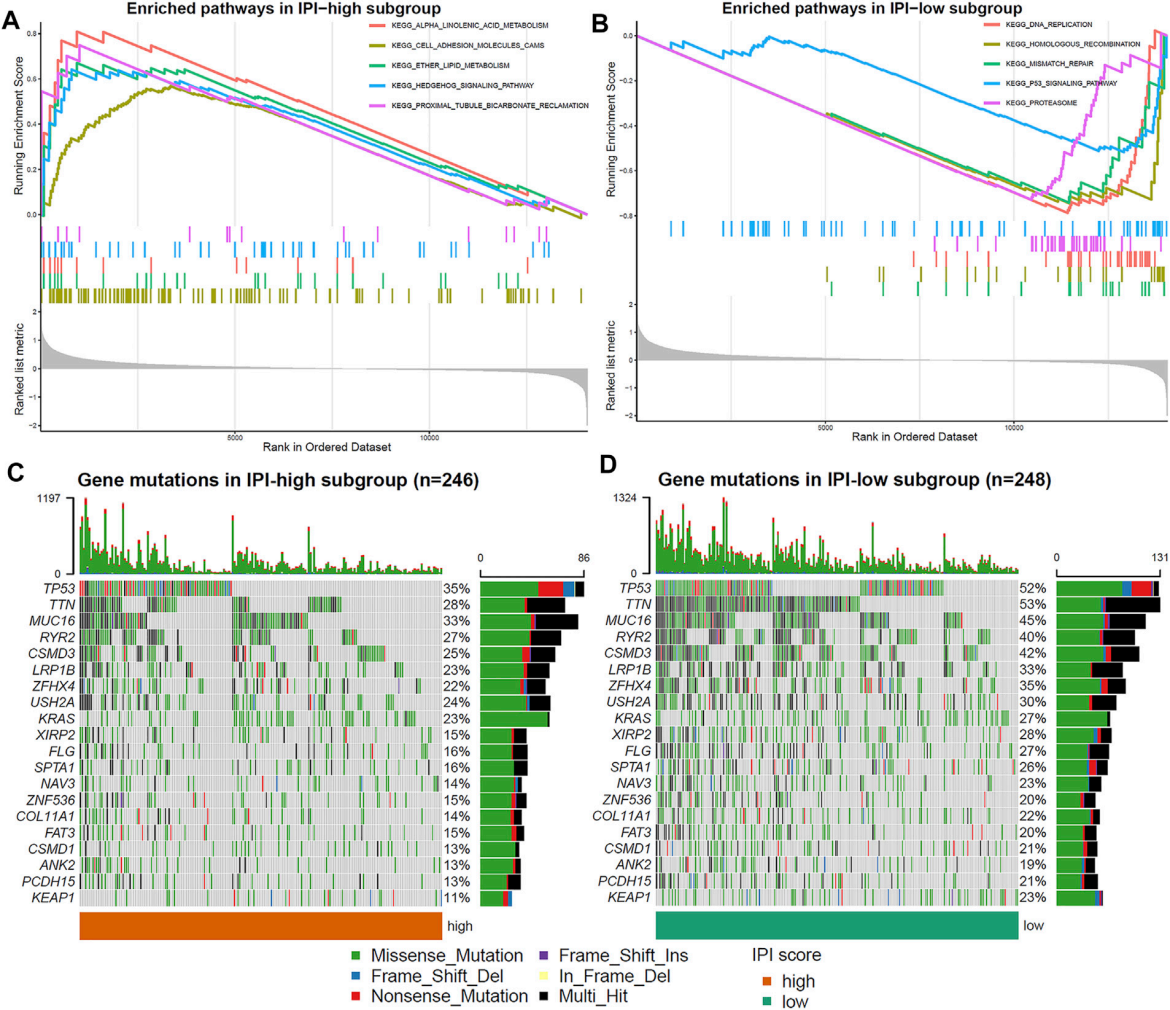
Molecular characteristics of IPI subgroups. **(A,B)** Enriched pathways in IPI-high and IPI-low subgroups (*p* < 0.05, FDR <0.25). **(C,D)** Top 20 gene mutations in IPI-high and IPI-low subgroups. The percentage of mutations is shown on the right and the total number of mutations on the top. Different colors represent different types of mutations.

GSEA was used to determine the differences of signal pathway between IPI subgroups. IPI-high subgroup was mainly enriched in cell adhesion molecule pathways and metabolism-related signaling pathways (*p* < 0.05, FDR <0.25; Figure 3A), and IPI-low subgroup was mainly enriched in proteasome and p53 signaling pathways (*p* < 0.05, FDR <0.25; Figure 3B). The detailed results of GSEA for the IPI subgroups were listed in Supplementary Table S6.

Next, in order to further understand the molecular characteristics of IPI, we analyzed the gene mutations of IPI subgroups. We showed the top 20 gene mutations in the IPI subgroups (Figures 3C,D). The most frequent mutation type was missense mutation, followed by nonsense mutation. The mutation rate of TP53, TTN, MUC16, RYR2, CSMD3, LRP1B, ZFHX4, USH2A and KRAS all exceeds 20% in IPI subgroups. In addition, the mutation rate of TP53, TTN and MUC16 in the IPI-low subgroup was significantly higher than that in the IPI-high subgroup.

### Immune Characteristics of Immune-Prognosis Index Subgroups

**FIGURE 4:**
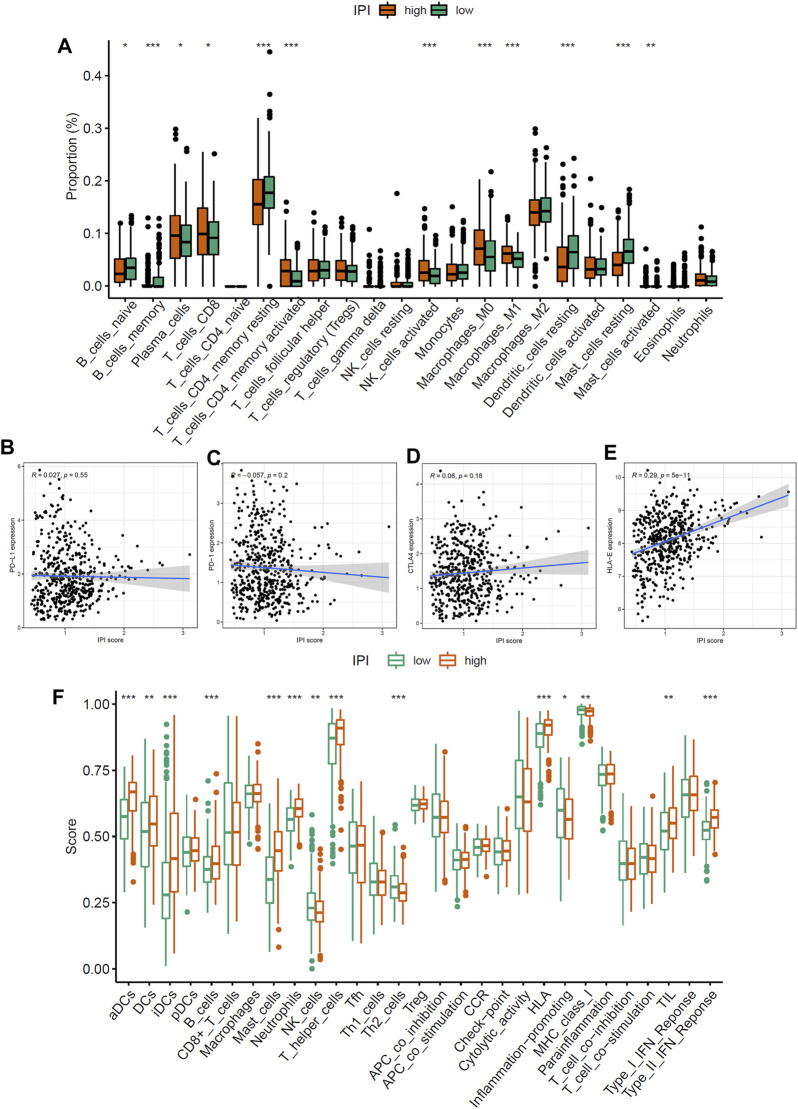
Immune characteristics of IPI subgroups. **(A)** The proportion difference of 22 immune cells in IPI subgroups in LUAD patients (*p < 0.05, **p < 0.01, ***p < 0.001). **(B–D)** The relationship between IPI scores and PD-1/PD-L1 and CTLA4 (*p* > 0.05). **(E)** The relationship between IPI scores and HLA-E (*p* < 0.05). **(F)** The difference of immune and molecular functions of IPI subgroups in LUAD patients (*p < 0.05, **p < 0.01, ***p < 0.001).

In order to determine the composition of immune cells in the IPI subgroups, we uploaded the sequencing data of LUAD to CIBERSORT to obtain the proportion of immune cells in the IPI subgroups. We found that CD8 T cells, plasma cells, activated memory CD4 T cells, dendritic cells and macrophages M1 were more abundant in IPI-high subgroup, while resting memory CD4 T cells, macrophages M2, resting dendritic cells and resting mast cells were more abundant in IPI-low subgroup (Figure 4A). By analyzing the correlation between different immune cells and IPI scores, we found that CD8 T cells, plasma cells, activated memory CD4 T cells, activated NK cells and macrophages M1 were positively correlated with IPI (Supplementary Figures S6A–F), while resting memory CD4 T cells, macrophages M2, resting dendritic cells and resting mast cells were negatively correlated with IPI (Supplementary Figure S6G–K). Next, we explored the relationship between IPI scores and PD-1/PD-L1, CTLA4, and HLA-E. We found that there was no correlation between PD-1/PD-L1 and CTLA4 and IPI scores (Figures 4B–D), while there was a significant correlation between HLA-E and IPI scores (*R* = 0.29, *p* < 0.001) (Figure 4E).

Next, we applied specific gene signature to define the immune and molecular functions of IPI subgroups. We found that activated dendritic cells, HLA, MHC class I and type II IFN response were more in IPI-high subgroup, while Th2 cells were more in IPI-low subgroup (Figure 4F). We further studied the prognostic value of IPI in different immune and molecular functions. We found that patients with more immune cell infiltrations and HLA have a better prognosis (Supplementary Figure S7). It could be seen that the more immune cell infiltrations in IPI-high subgroup, the better prognosis in LUAD patients.

### Relationship Between Immune-Prognosis Index and Clinical Characteristics and Immune Subtype

**FIGURE 5:**
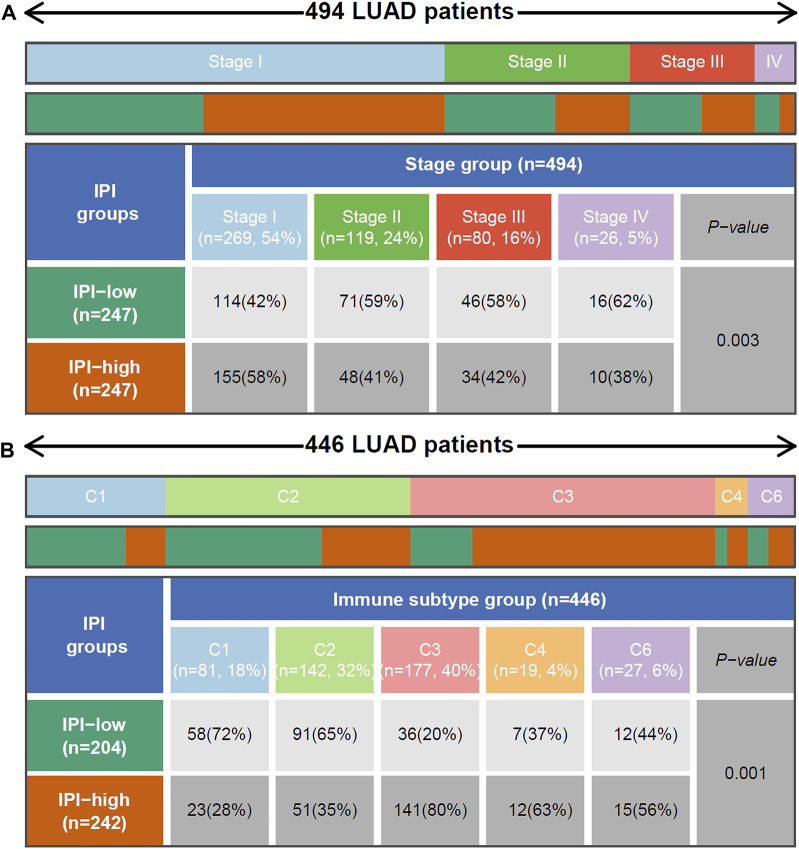
Relationship between IPI and tumor stage and reported immune subtype. **(A)** Heat map and table showing the distribution of LUAD tumor stage between IPI subgroups. **(B)** Heat map and table showing the distribution of LUAD immune subtype (C1, C2, C3, C4 and C6) between IPI subgroups. C1: Wound Healing, C2: IFN-γ Dominant, C3: Inflammatory, C4: Lymphocyte Depleted and C6: TGF-β Dominant. The distributions of tumor stage and reported immune subtype in the IPI subgroups were compared through the chi-square test.

The 494 LUAD patients of TCGA were grouped by tumor stage. We found that from stage I to stage IV, the percentage of patients in the IPI-high subgroup gradually decreased, while the percentage in the IPI-low subgroup gradually increased. Among them, patients with stage IV in the IPI-low subgroup (62%) were more than IPI-high subgroup (38%) (Figure 5A). According to the pathological subtype, 501 LUAD patients were divided into 6 groups. As shown in Supplementary Figure S8, solid tumors only exist in the IPI-low subgroup, and there were more mucinous and papillary LUAD in the IPI-high subgroup (*p* < 0.001, chi-square test).

According to the reported method of immunotype, the immune types of all samples can be divided into 6 types (C1-C6): Wound Healing, IFN-γ Dominant, Inflammatory, Lymphocyte Depleted, Immunologically Quiet, and TGF-β Dominant (Thorsson et al., 2018). From Figure 5B, we found that there were more Inflammatory (C3) in the IPI-high subgroup, while there were more Wound Healing (C1) in the IPI-low subgroup. The survival of C3 patients is significantly better than that of TGF-β Dominant (C6) patients (Supplementary Figure S9A, *p*=0.0244). At the same time, according to the IPI grouping, we could see that among the C3 patients, the patient’s prognosis in the IPI-high group was better than in the IPI-low group (Supplementary Figure S9B, *p*=0.0465); in the C6 patients, there was no significant difference between IPI high and low groups, but there was still a trend of better prognosis in the IPI-high group (Supplementary Figure S9C).

### The Benefit of Anti-PD-1 Therapy in Different Immune-Prognosis Index Subgroups

**FIGURE 6:**
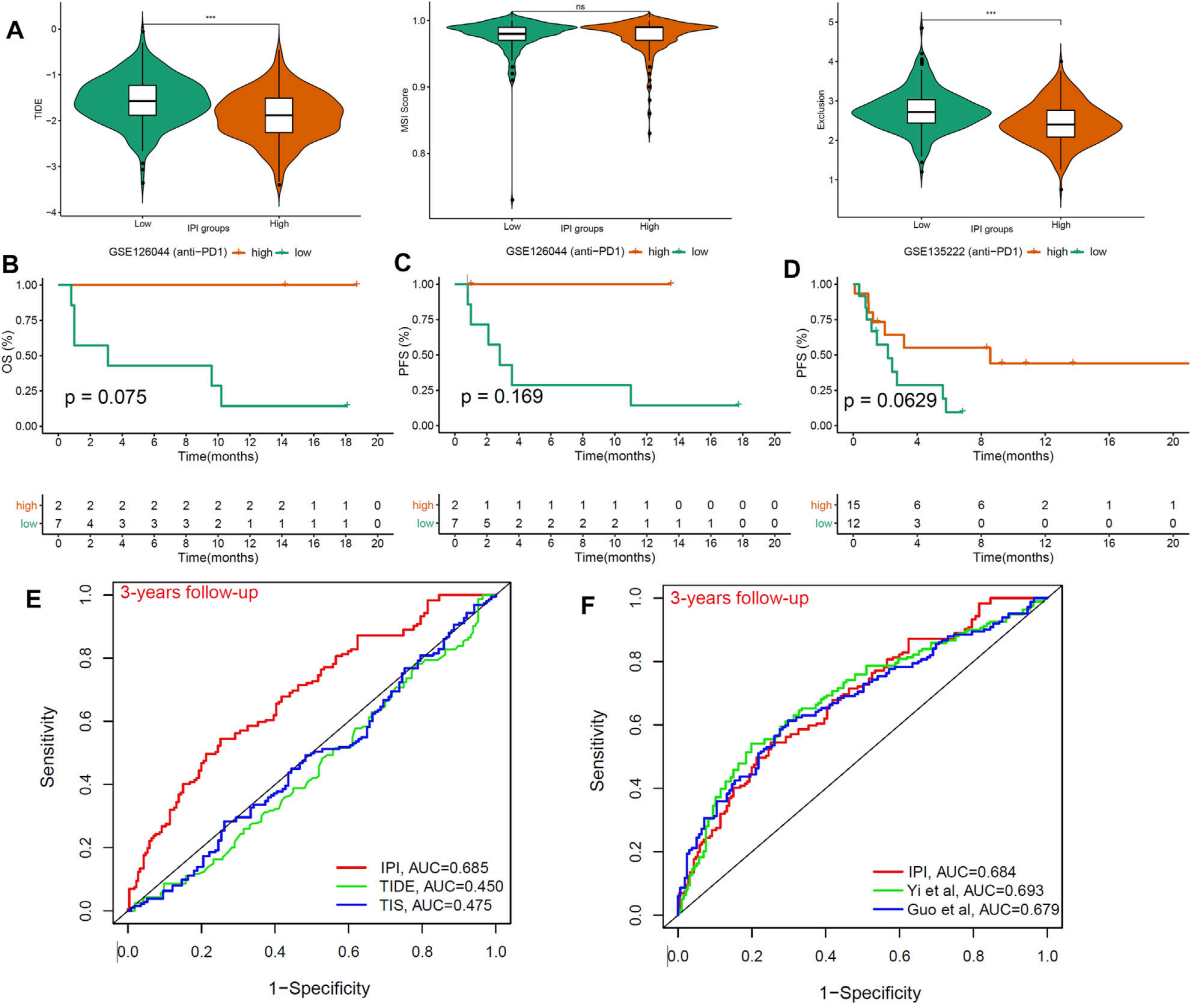
The benefit of IPI in LUAD patients with anti-PD1 therapy. **(A)** Compared the scores of TIDE, MSI, and T cell exclusion and dysfunction in two IPI subgroups (ns: not significant, *p < 0.05, **p < 0.01, ***p < 0.001). **(B,C)** Kaplan-Meier survival analysis of IPI subgroups in the GSE126044 (*n* = 9) with anti-PD-1 therapy. **(D)** Kaplan-Meier survival analysis of IPI subgroups in the GSE135222 (*n* = 27) with anti-PD-1 therapy. **(E)** ROC analysis of IPI, TIDE and TIS on OS at 3-years follow-up in LUAD patients. **(F)** ROC analysis of IPI, and other two models on OS at 3-years follow-up in LUAD patients.

We used TIDE to score T cell dysfunction in samples from different IPI subgroups and predict their response to ICB treatment (Jiang et al., 2018). A higher TIDE score indicates a higher probability of immune escape, which suggested that patients were less likely to benefit from immunotherapy. In our results, the IPI-high subgroup has a lower TIDE prediction score compared to IPI-low subgroup (Figure 6A). Meanwhile, we found that IPI-low subgroup had a higher T cell exclusion scores, but there was no difference in microsatellite instability (MSI) score between two IPI subgroups (Figure 6A). In addition, we also evaluated the prognostic value of IPI in two LUAD cohorts (GSE126044 and GSE135222) with anti-PD-1 treatment. We found that compared with IPI-low subgroup, patients in the IPI-high subgroup have better OS and PFS (Figures 6B–D). In summary, patients in IPI-high subgroup have less immune escape and better prognosis than IPI-low subgroup.

Finally, we compared the prognostic value of IPI, TIDE and TIS in different follow-up times in LUAD patients. The result showed that the AUC for IPI (AUC = 0.685) were better at 3-years follow-up than TIDE and TIS (AUC = 0.45, 0.475) (Figure 6E). At the same time, compared with the other two reported models, IPI still had good predictive value (AUC = 0.684) (Figure 6F). Therefore, we recommended using IPI to predict the prognosis of LUAD patients, especially those receiving ICB treatment.

## 4 Discussion

Lung cancer remains one of the most common malignant tumors worldwide (Yoda et al., 2019). With the improvement of surgical techniques, the continuous optimization of traditional treatments and the development of immunotherapy, the overall survival rate of lung cancer has been significantly improved (Petrella and Spaggiari 2016). In recent years, ICB treatment has been proven to be an effective treatment for advanced LUAD and improve patient’s survival (Shah et al., 2019; Garassino et al., 2020). Since the overall response rate of ICB treatment is still low (Ferris et al., 2016; Larkins et al., 2017), it is particularly important to determine which patients could benefit from ICB treatment. However, there is still less hopeful candidate biomarkers which can be used to predict the response of ICB treatment. This also highlights the importance of finding prognostic biomarkers for ICB treatment in LUAD.

Network analysis is different from conventional difference analysis, which could help us to detect key node factors related to event occurrence from omics data (Puga et al., 2015). In our study, based on transcriptome data of LUAD and immune gene sets, we have used correlation and causal network analysis to identify 15 immune hub genes, and constructed IPI with 5 immune-prognosis genes (A2M, ADRB1, ADRB2, VIPR1 and PTH1R). Combined with clinical characteristics, IPI was proved to be an independent prognostic factor for LUAD (*p* = 0.008). Our results showed that there were active immune response and better survival in the IPI-high subgroup, while immune-suppressive response and worse survival in the IPI-low subgroup.

IPI was made up of the following five genes, A2M, ADRB1, ADRB2, VIPR1 and PTH1R. α2-macroglobulin (A2M) is known as an acute phase protein of the innate immune system. It is a general protease inhibitor in serum and can bind a variety of cytokines and growth factors (Mocchegiani and Malavolta 2007; Xue et al., 2017). Taking advantage of the immunoaffinity of A2M protein complex in human serum, more and more studies have taken A2M protein complex as a new cancer serum biomarker (Kanoh et al., 2001; Burgess et al., 2008). As for β1 and β2 adrenergic receptors (ADRB1/ADRB2), the adrenergic receptors (AR) are a prototypic family of guanine nucleotide binding regulatory protein-coupled receptors that mediate the physiological effects of the hormone epinephrine and the neurotransmitter norepinephrine. Studies have shown that the systemic activation of *β*-AR (β1+β2) *in vivo* can enhance the mobilization, expansion, and anti-tumor activity of TCR-gamma delta T cells (Baker et al., 2019). As for vasoactive intestinal polypeptide receptor 1 (VIPR1), it is the receptor of VIP which can be considered as an anti-inflammatory and immunomodulatory agent (Gomariz et al., 2006). VIP can increase the differentiation of Th2 and T regulatory (Treg) subsets and reduce the differentiation of Th1 and pathogenic Th17 cells (Delgado et al., 2001; Jimeno et al., 2014). In addition, studies have reported the healing effects of VIP in animal models of inflammatory/autoimmune diseases (Abad et al., 2003; Gonzalez-Rey et al., 2007). According to the formula of IPI, A2M was negatively associated with IPI, while ADRB1, ADRB2, VIPR1 and PTH1R were positively associated with IPI, and HR < 1. It can be seen that higher IPI score indicates better prognosis, lower IPI score indicates worse prognosis.

In order to understand the molecular characteristics between different IPI subgroups, we first analyzed gene mutations in each subgroup. The most common mutation was missense mutation, then nonsense mutation, consistent with previous reports (Martincorena and Campbell 2015). The mutations of TP53 and TTN in the IPI-low subgroup were more common than in the IPI-high subgroup (TP53: 52% *vs*. 35%; TTN: 53% *vs*. 28%). TP53 was identified as the most frequently mutated gene in cancers. As a transcription factor, p53 is involved in a variety of cellular processes, including DNA repair, cell cycle arrest, senescence, and apoptosis (Bieging et al., 2014). About half of cancer patients have TP53 mutation, and its mutations have been confirmed to be associated with poor prognosis in many cancers, especially in LUAD (Olivier et al., 2010; Skoulidis and Heymach 2019). Besides, TTN is also frequently mutated in a variety of cancers, including lung squamous cell carcinoma, LUAD and colon adenocarcinoma (Kim et al., 2017). Studies have shown that the mutation load within TTN represents high TMB status (Oh et al., 2020). Therefore, IPI-low patients with high TP53 and TTN mutations have a worse prognosis than IPI-high patients with low TP53 and TTN mutations, in consistent with our survival results.

Understanding the composition of immune cells in tumor tissue will help to find new method of cancer treatment and improve the efficiency of ICB treatment. CD8 T cells, activated memory CD4 T cells, activated NK cells and macrophages M1 were more abundant in IPI-high subgroup, while resting memory CD4 T cells, macrophages M2 and resting dendritic cells were more abundant in IPI-low subgroup. A lot of research have shown that the dense infiltrations of CD8 T cells and macrophages M1 indicate a favorable prognosis (Gentles et al., 2015; Fridman et al., 2017; Garrido-Martin et al., 2020). NK cells can enhance the response of antibodies and T cells to tumors, and improve the prognosis of patients (Shimasaki et al., 2020; Myers and Miller 2021). In addition, macrophages M2 is proved to be related to tumor growth and the development of aggressive phenotypes, as well as the poor prognosis of gastric cancer, breast cancer, bladder cancer, prostate cancer and lung cancer (Ruffell and Coussens 2015; Pathria et al., 2019). Our results also confirmed that IPI-high subgroup with more infiltrations of CD4 T cells, NK cells and macrophages M1 have a better prognosis, while IPI-low subgroup with more infiltrations of macrophages M2 have a poor prognosis.

Next, we explored the relationship between IPI scores and ICB targets (PD-1/PD-L1 and CTLA4) and HLA-E. It has been proven that PD-L1^+^ tumors have better response to anti-PD-1/PD-L1 therapy than PD-L1^-^ tumors (Hansen and Siu 2016; Oliva et al., 2019). However, our results showed that there was no correlation between IPI and PD-1/PD-L1 and CTLA4. We believe that the unclear threshold for the positive expression of PD-1/PD-L1 and CTLA4 in the LUAD transcriptome data may be the main reason for this negative result. Moreover, based on the immunohistochemistry to detect the expression of PD-1/PD-L1 and CTLA4 is more valuable than transcriptome sequencing. Therefore, further research is needed to clarify the correlation between IPI and PD-1/PD-L1 and CTLA4. Besides, our results showed that IPI were positively correlated with HLA-E (*p* < 0.001). HLA-E is mainly expressed on the surface of endothelial cells, T and B lymphocytes, monocytes and macrophages, and is a presentation molecule for endogenous antigens (Kochan et al., 2013). Studies have found that HLA-E antigen peptide complexes can limit the cytotoxicity of CD8^+^ T cells and play an important regulatory role in adaptive immunity (Pietra et al., 2010). And we found that there was more HLA and MHC class I in the IPI-high subgroup, which is also consistent with the above results. These results indicated that there were a large number of MHC class I molecules in the IPI-high subgroup, which have the characteristics of immune activation and the inhibition of tumor progression.

IPI grouping could distinguish different stages, pathological subtypes and reported immune subtype. In addition, according to Thorsson’s study, the immune landscape of cancer have significant differences in macrophage, extent of intratumoral heterogeneity, aneuploidy, extent of neoantigen load, overall cell proliferation, expression of immunomodulatory genes, and prognosis (Thorsson et al., 2018). And high content of C3 (inflammatory) can promote tumor immunity, and high content of C1 (Wound Healing) and C6 (TGF-β dominant) can inhibit tumor immunity (Lee and Margolin 2011; Nakamura and Smyth 2017). These studies were consistent with our results, that is, the IPI-low subgroup was characterized by more advanced, solid and C1, immune-suppressive response and worse prognosis in LUAD patients, while the IPI-high subgroup was characterized by more early, mucinous, papillary and C3, active immune response and better prognosis in LUAD patients.

The difference of T cell function between IPI subgroups identified by TIDE could reflect the effectiveness of ICB treatment (anti-PD-1/PD-L1 and anti-CTAL4). In our study, patients with IPI-low subgroup had higher scores of TIDE and T cell exclusion. It can be seen that the low response of ICB treatment in IPI-low subgroup may be caused by immune escape via T cell exclusion. The “T cell dysfunction” in the TIDE prediction score represents the proportion of inactive T cells in the tumor (Jiang et al., 2018). IPI-low subgroup had a higher T cell dysfunction score than IPI-high subgroup. This result suggested that there were a lot of T cell infiltrations in the IPI-low subgroup samples, but most of them were inactive. The expression of T cell failure markers leads to the inactivation of T cells in the tumor microenvironment may be the reason for this result (Chen et al., 2019). In order to further verify the prognostic value of IPI, we performed survival analysis on the LUAD patients within anti-PD-1 treatment. We found that the prognosis of patients in the IPI-high subgroup was better than IPI-low subgroup, indicating that IPI could effectively distinguish the outcomes in LUAD patients with anti-PD-1 treatment.

Some identified biomarkers, such as TIDE and TIS, can predict the response to immunotherapy. Both TIDE and TIS mainly reflect the functional status of T cells, and cannot fully reflect the changes in the tumor immune microenvironment during ICB treatment (Ayers et al., 2017; Jiang et al., 2018). In addition, these two biomarkers were mainly used to predict the patient’s response to immunotherapy, but survival time was also important when making treatment decision. In our study, IPI could predict the response of ICB treatment and was a good predictor for long-term follow-up than TIDE and TIS. Moreover, IPI has only five genes, which was easier to detect than TIDE and TIS.

In summary, IPI is a potentially valuable immune-prognosis biomarker. IPI grouping helps distinguish the anti-tumor immune status in LUAD patients.

## Data Availability

The datasets analyzed during this research are available in TCGA (http://cancergenome.nih.gov/) and GEO (https://www.ncbi.nlm.nih.gov/geo, GSE30219, GSE37745, GSE68465, GSE126044 and GSE135222) data bases. The data are also available from the corresponding author (senyi_deng@scu.edu.cn) on reasonable request.
