# Supplementary material for: A Novel Immune-Prognosis Index Predicts the Benefit of Lung Adenocarcinoma Patients
Source: Front Pharmacol. 2022 May 9;13:818170. doi: 10.3389/fphar.2022.818170 (PMC9124834; doi:10.3389/fphar.2022.818170)
Supplement: Supplementary file 7 [file Image1.pdf]

frontiers

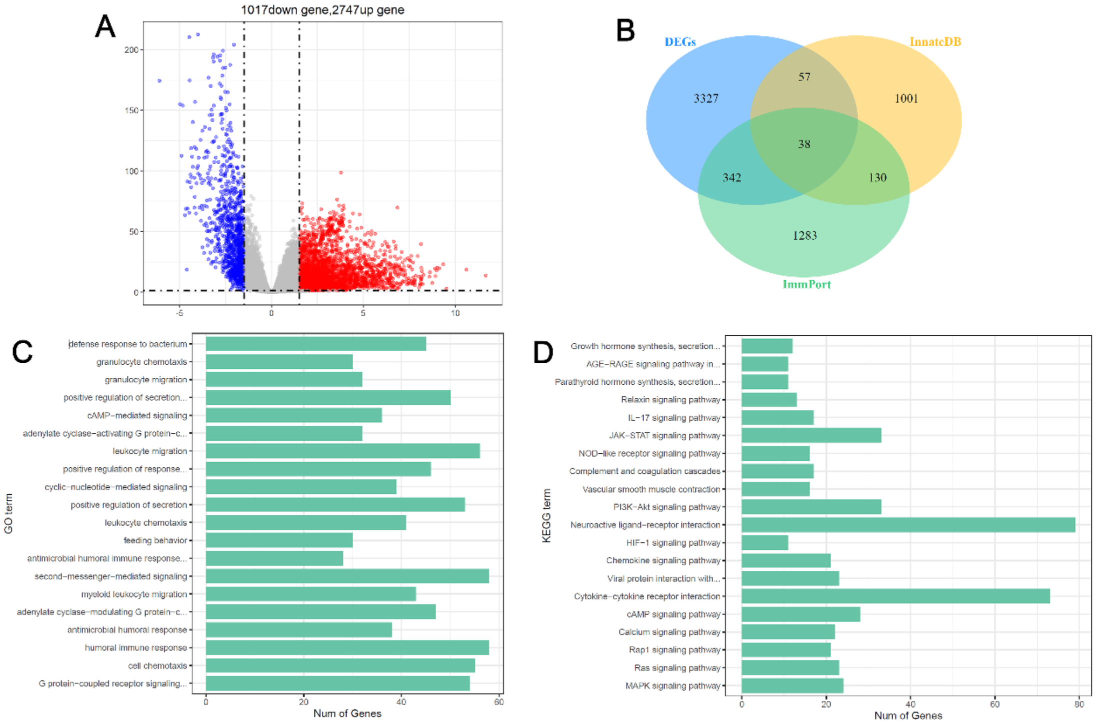

Figure S1 Identify differential immune genes of LUAD. (A) Differential expression analysis between 535 tumors *vs* 59 normal lung tissues of LUAD patients ($p < 0.05$, $|\log2FC| > 1.5$). (B) Intersecting differential genes with immune gene sets obtained from ImmPort and InnateDB. (C) Gene Ontology (GO) enrichment analysis of the differential immune genes ($p < 0.05$). (D) Kyoto Encyclopedia of Genes and Genomes (KEGG) pathway analysis of the differential immune genes ($p < 0.05$). DEGs: differential expression genes.

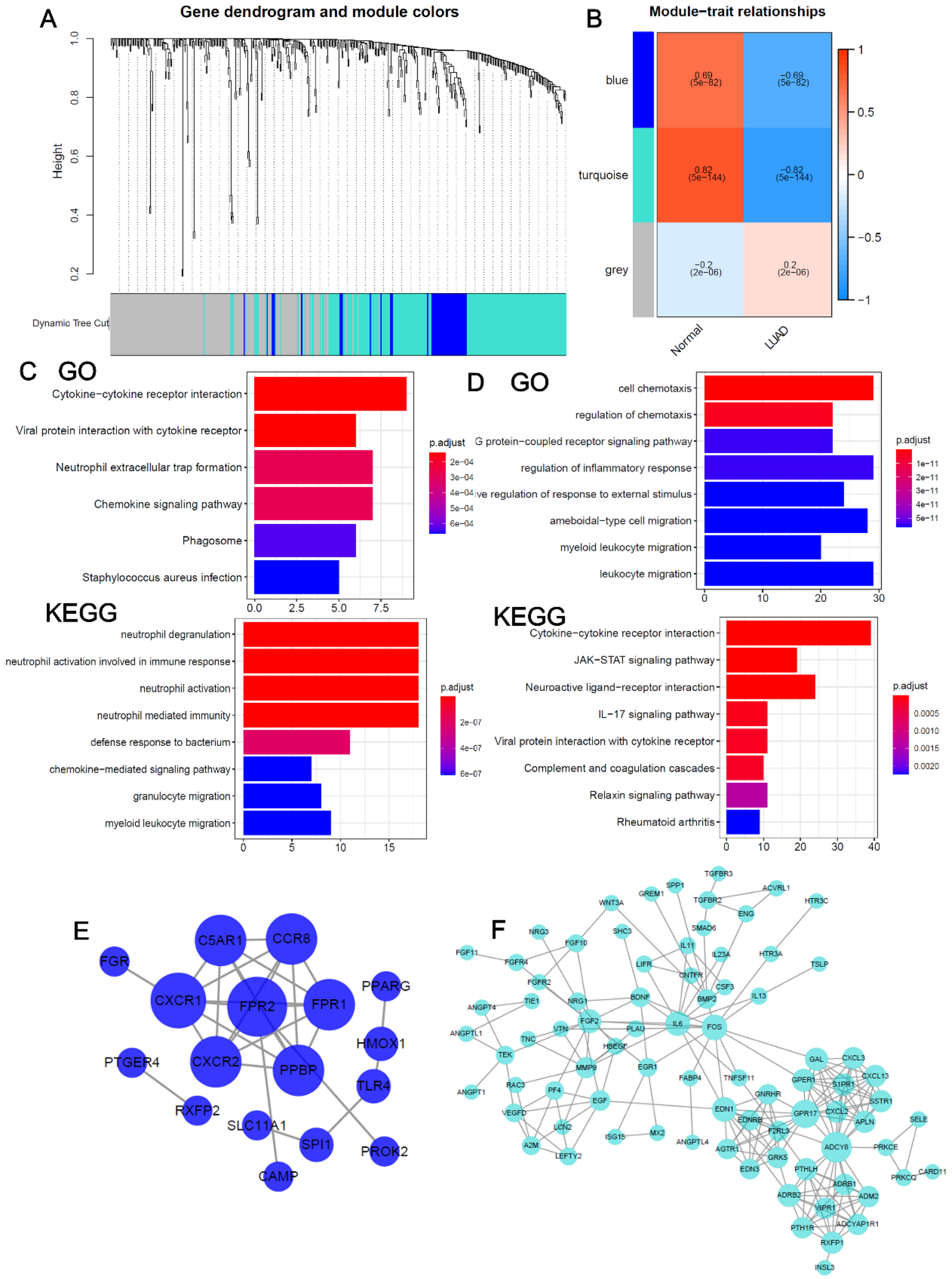

Figure S2 WGCNA identifies LUAD-related immune gene modules. (A) Weighted gene co-expression network analysis (WGCNA) of differential immune genes with a soft threshold β = 5. (B) Obtain gene modules related to LUAD based on the analysis of Pierson correlation. (C) The top 8 GO terms and KEGG pathways of genes in the blue module were shown ($p < 0.05$). (D) The top 8 GO terms and KEGG pathways of genes in the turquoise module were shown ($p < 0.05$). (E) The

network relationship of genes in the blue module (combine-score > 0.9). (F) Correlation network of genes in the turquoise module (combine-score > 0.9).

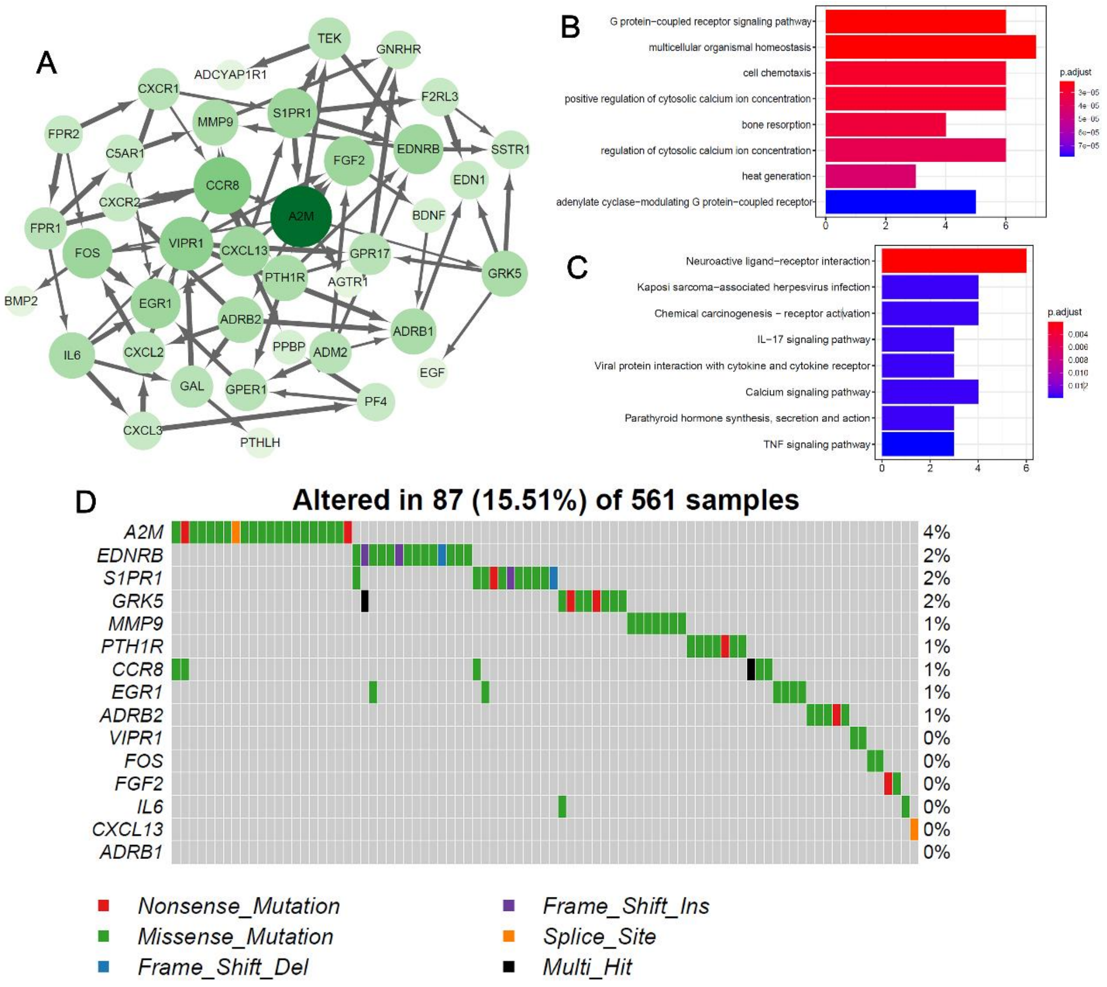

Figure S3 Causality network analysis to determine immune hub genes of LUAD. (A) Based on the degree >5 of correlation network genes, the causal network of 39 immune genes were shown (gene strength > 0.8). (B, C) The GO and KEGG function enrichment analysis of 15 immune hub genes ($p < 0.05$). (D) The mutation rate of 15 immune hub genes were displayed.

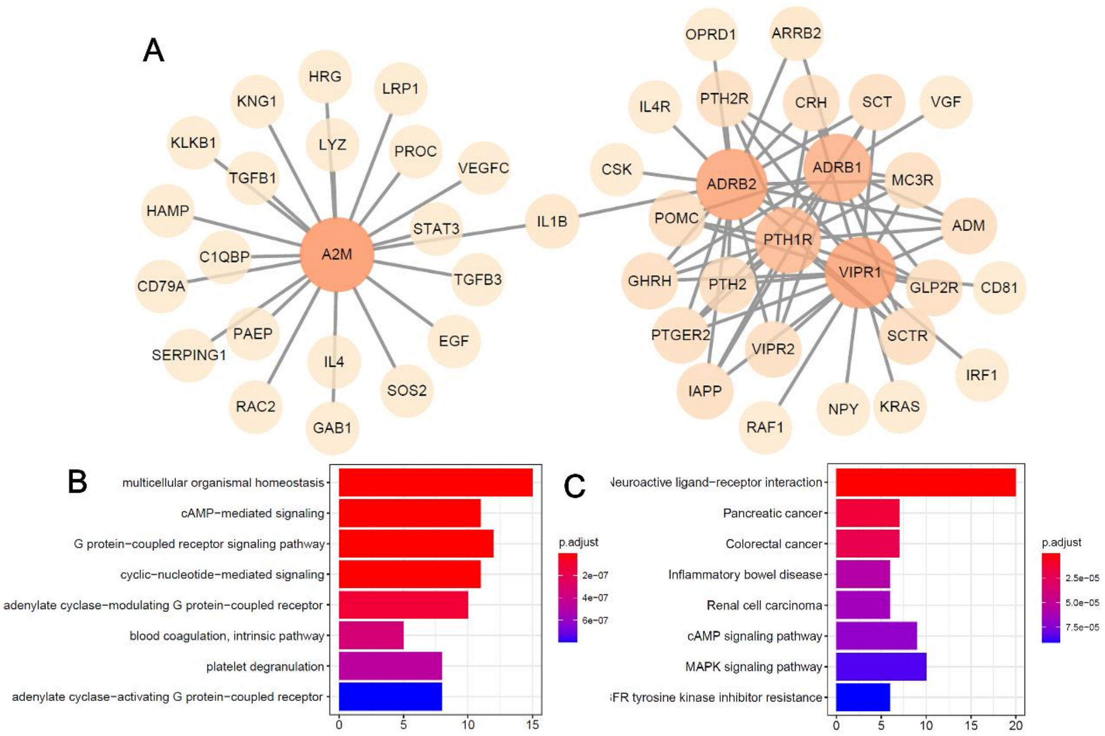

Figure S4 The correlation and biological functions of 5 immune-prognosis hub genes. (A) The correlation network between 5 immune-prognosis hub genes and immune genes. (B, C) The GO and KEGG function enrichment analysis of5 immune-prognosis hub genes ($p < 0.05$).

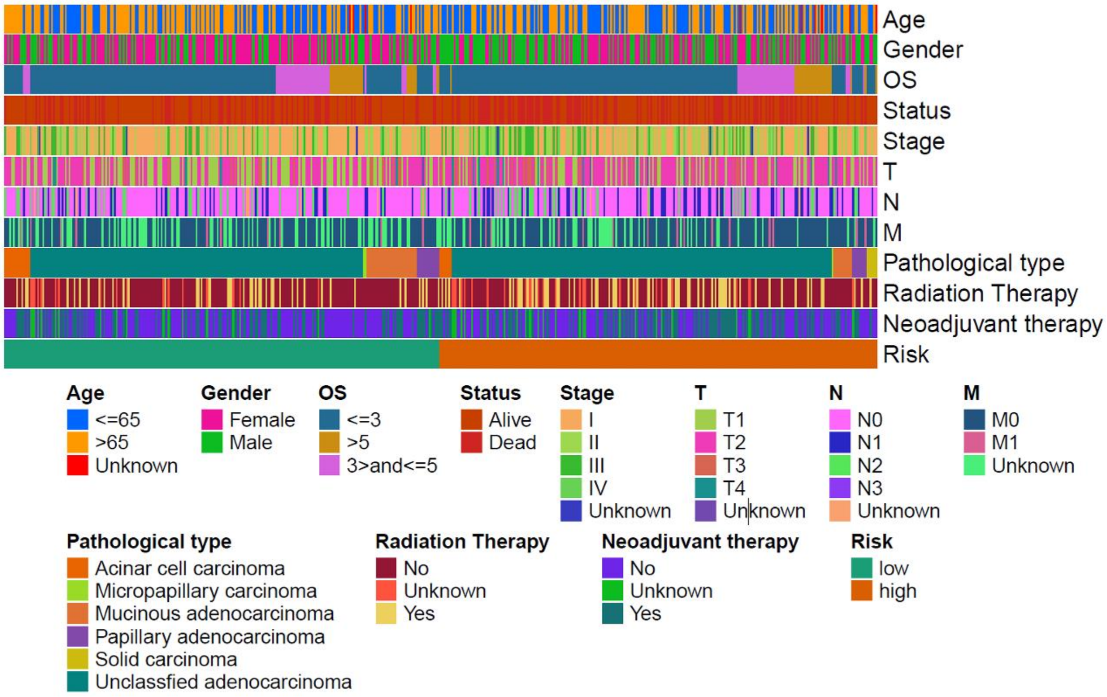

Figure S5 Detailed clinical characteristics of LUAD patients in TCGA cohort. Between IPI subgroups, these clinical characteristics were distributed equally ($p > 0.05$). Age, Gender, Overall

survival, Survival status, Tumor stage, T stage, N stage, M stage, Pathological type, Radiation therapy and Neoadjuvant therapy were shown as patient annotations.

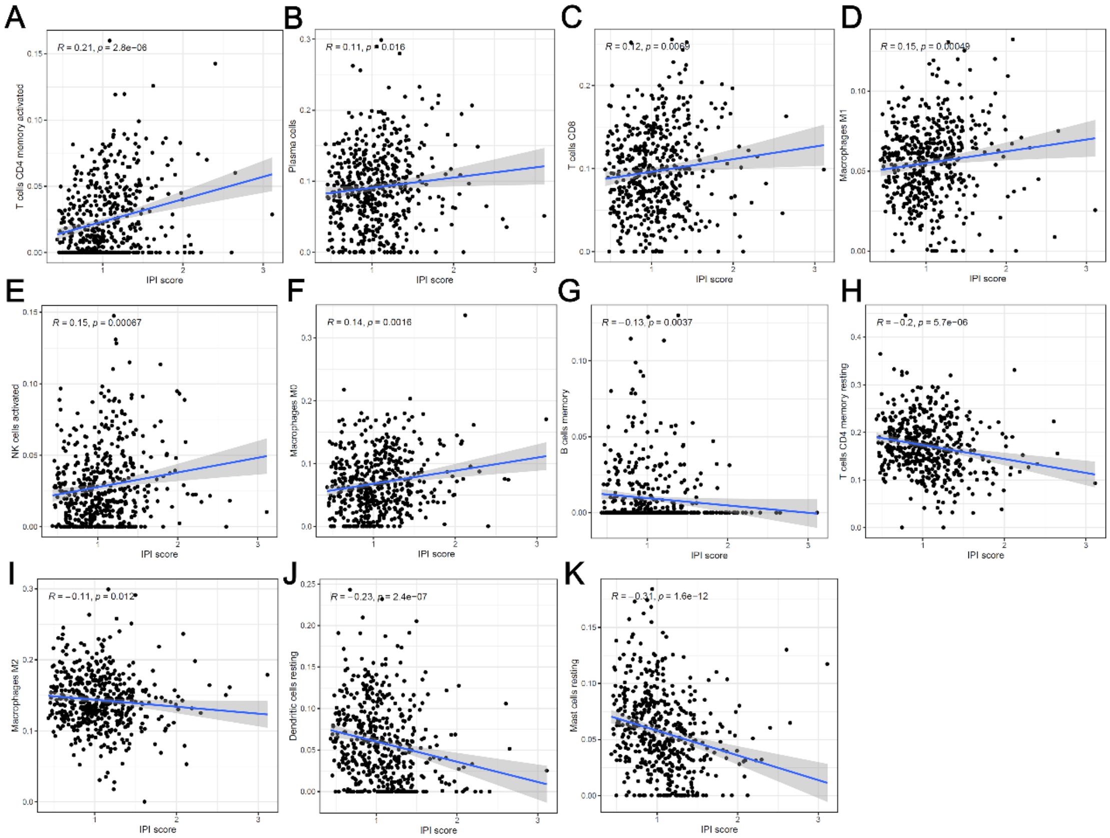

Figure S6 The relationship between different immune cell infiltrations and IPI scores. (A-F) CD8 T cells, plasma cells, activated memory CD4 T cells, activated NK cells and macrophages M1 were positively correlated with IPI scores ($p < 0.05$). (G-K) Resting memory CD4 T cells, macrophages M2, resting dendritic cells and resting mast cells were negatively correlated with IPI scores ($p < 0.05$).

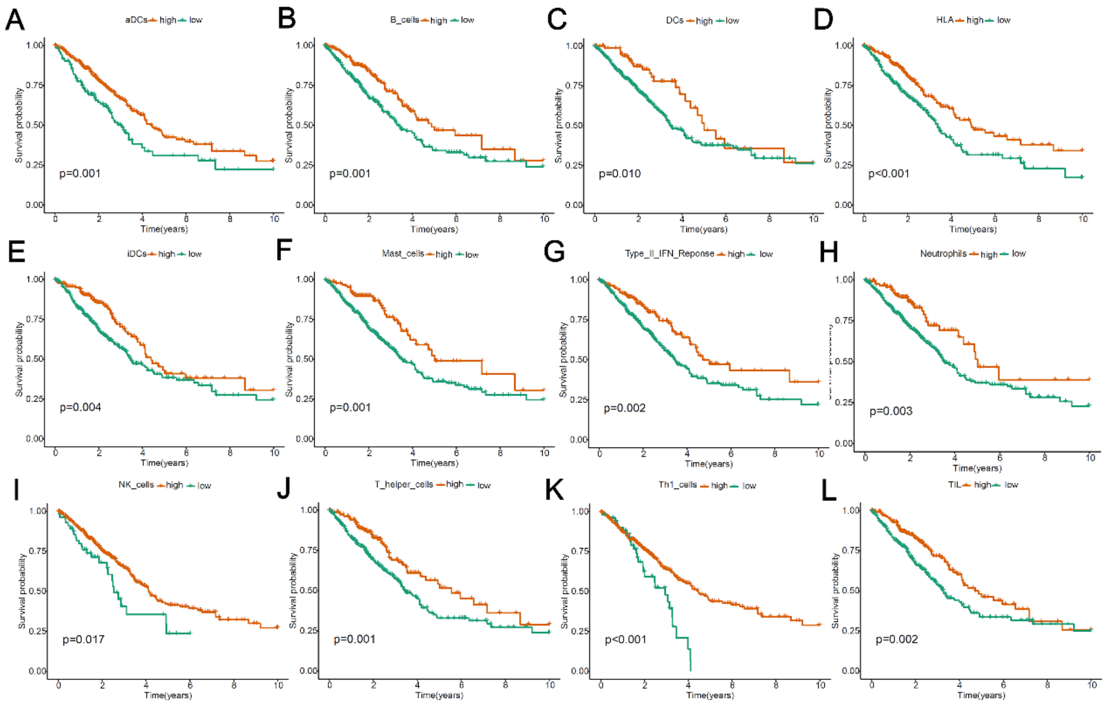

Figure S7 Kaplan-Meier survival analysis of the different immune and molecular functions in IPI subgroups.

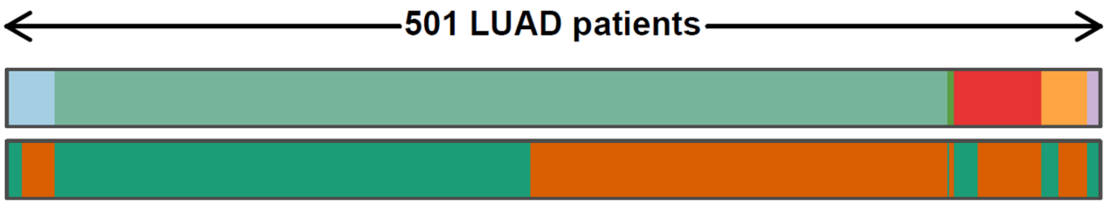

IPI groups	Pathological subtype group (n=501)						
	Acinar (n=22, 4%)	Adenocarcinoma (n=409, 82%)	Micropapillary (n=3, 1%)	Mucinous (n=40, 8%)	Papillary (n=21, 4%)	Solid (n=6, 1%)	*P*-value
IPI-low (n=251)	7(32%)	218(53%)	1(33%)	11(28%)	8(38%)	6(100%)	0.001
IPI-high (n=250)	15(68%)	191(47%)	2(67%)	29(72%)	13(62%)	0(0%)	

Figure S8 Relationship between IPI and pathological subtype. Heat map and table showing the distribution of LUAD pathological subtype (acinar, adenocarcinoma, micropapillary, mucinous, papillary and solid) between IPI subgroups. The distributions of pathological subtype in the IPI subgroups were compared through the chi-square test.

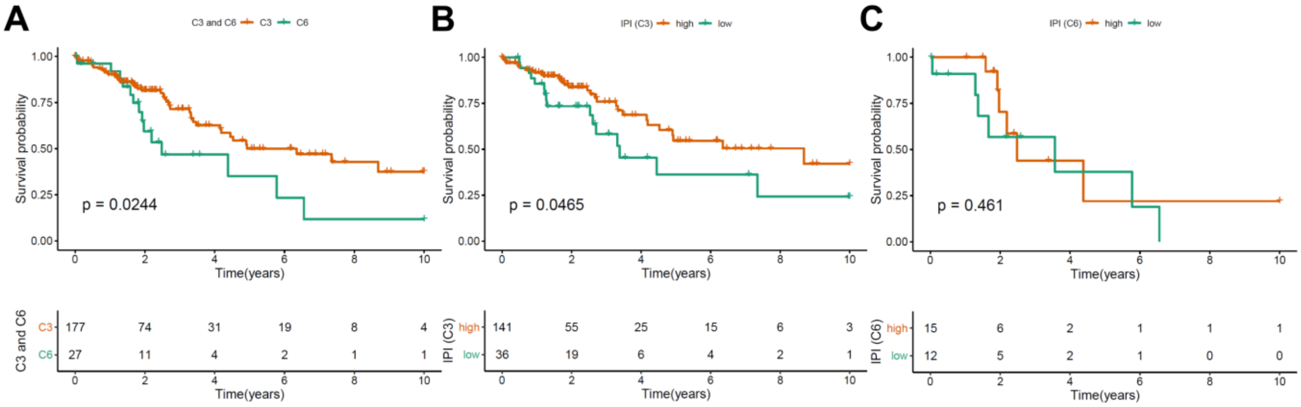

Figure S9 Kaplan-Meier survival analysis of the Inflammatory (C3) and TGF-β Dominant (C6) in IPI subgroups.
